# Case Report: Diagnosis and treatment of equine ascending placentitis: compilation of 17 case reports

**DOI:** 10.3389/fvets.2025.1591452

**Published:** 2025-06-18

**Authors:** Elisabeth Hemberg, Jane M. Morrell

**Affiliations:** ^1^Haddebo Seminstation, Hjortkvarn, Sweden; ^2^Department of Clinical Sciences, Swedish University of Agricultural Sciences, Uppsala, Sweden

**Keywords:** case report, equine placentitis, premature udder activity, vulval discharge, vulval conformation

## Abstract

Placentitis (inflammation of the placenta) most commonly occurs during the last trimester of pregnancy, frequently due to bacterial entry via the vulva. The outcome of the pregnancy, i.e., prevention of abortion or the birth of a compromised foal, depends on when treatment is initiated, the appropriate medication, and surgical correction of the vulva to ensure an effective seal. In this study, during the period 2012–2024, 17 mares were referred to the clinic, presenting with signs of placentitis, most commonly premature udder activity and/or discharge from the vulva. All mares maintained the pregnancy after treatment, ultimately producing live foals that survived. The earliest cases involved mares with the most pronounced clinical signs; these mares received treatment with poor perfusion into the placenta and delivered septicaemic foals. The remaining foals born exhibited only minor clinical signs or appeared healthy. For later cases, treatment with drugs providing good uterine perfusion continued until foaling, including trimethoprim sulphate twice a day and acetylsalicylic acid, a COX-1 anti-inflammatory drug, administered twice a day until parturition, if necessary. In addition, for 16 mares, a vulvoplasty (Caslick’s operation) was performed, or was extended if the mare had already undergone surgery.

## 1 Introduction

Placentitis is inflammation of the placenta, most commonly due to a bacterial infection (1–3). The normal cervix and the lower reproductive tract, which includes the vagina, the vestibule, and the vulva (4), provide a physical barrier to the entry of foreign microorganisms to the foetoplacental unit (5). In ascending placentitis, the infection is caused by pathogens gaining entry through the cervix (3). There are different types, depending on morphology and pathogenesis: ascending via the vulva, haematogenous, and nocardioform (US). Regardless of the cause, the infection results in an inflammatory reaction in the placenta and eventually in the foetus. The most common cause is an ascending infection, with bacteria entering via the vulva (5), continuing through the cervical canal into the placenta and foetus.

**Figure 1:**
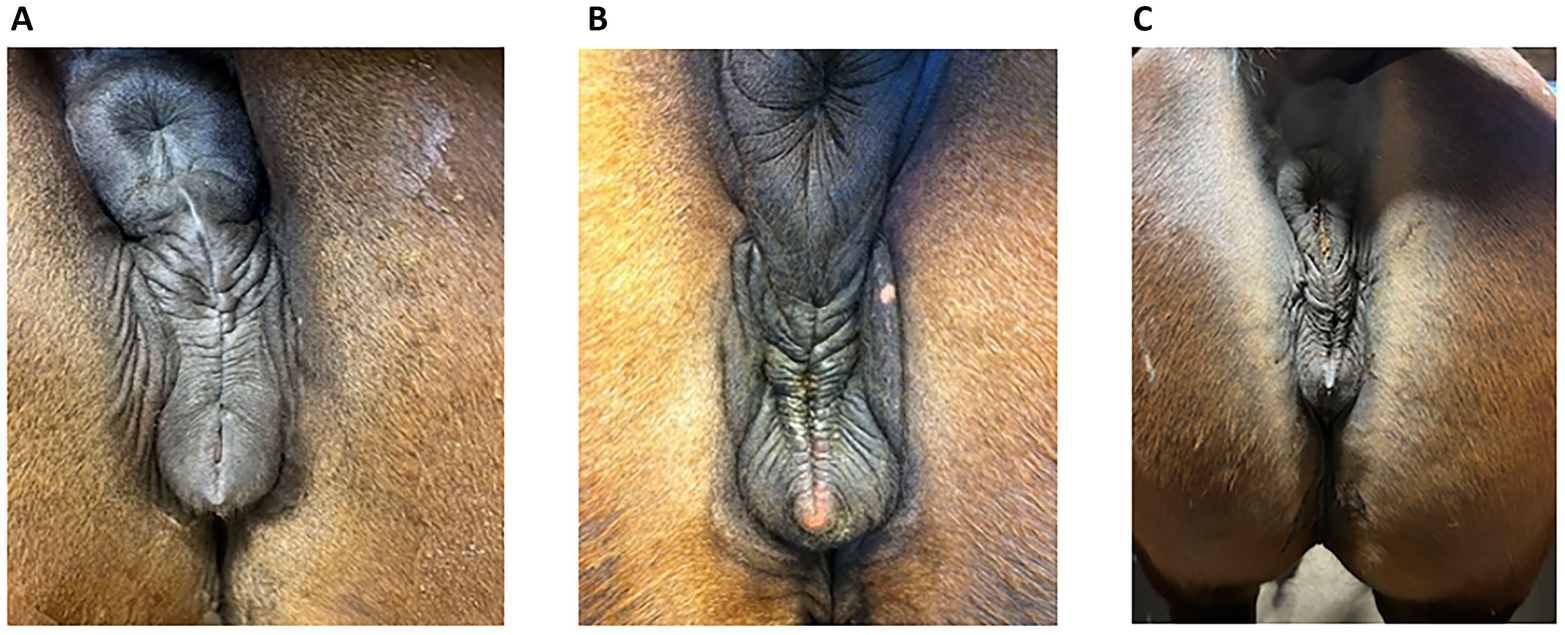
Some examples of vulval conformation from mares presenting with placentitis in late pregnancy. **(A)** The mare described in Case 16, at day 310 of gestation. The vulval plicae are angular and uneven, are not horizontal and do not have a uniform distance to each other from the top to the pelvic brim. **(B)** The mare in Case 17 at day 302 of gestation. The vulval plicae are more pronounced, showing the angular and uneven pattern clearly. **(C)** An 8-year-old trotting mare (from 2025) at day 264, CTUP 11 mm, detachment of placenta, soft cervix, red discharge from vulva and an active udder. This vulval pattern has only minor angulation and unevenness, making the decision of the vulva sealing questionable, but the other clinical signs were indicative of a diagnosis of placentitis. The clinical signs disappeared on treatment. This mare has not foaled yet and is therefore not included in the case report.

**Figure 2:**
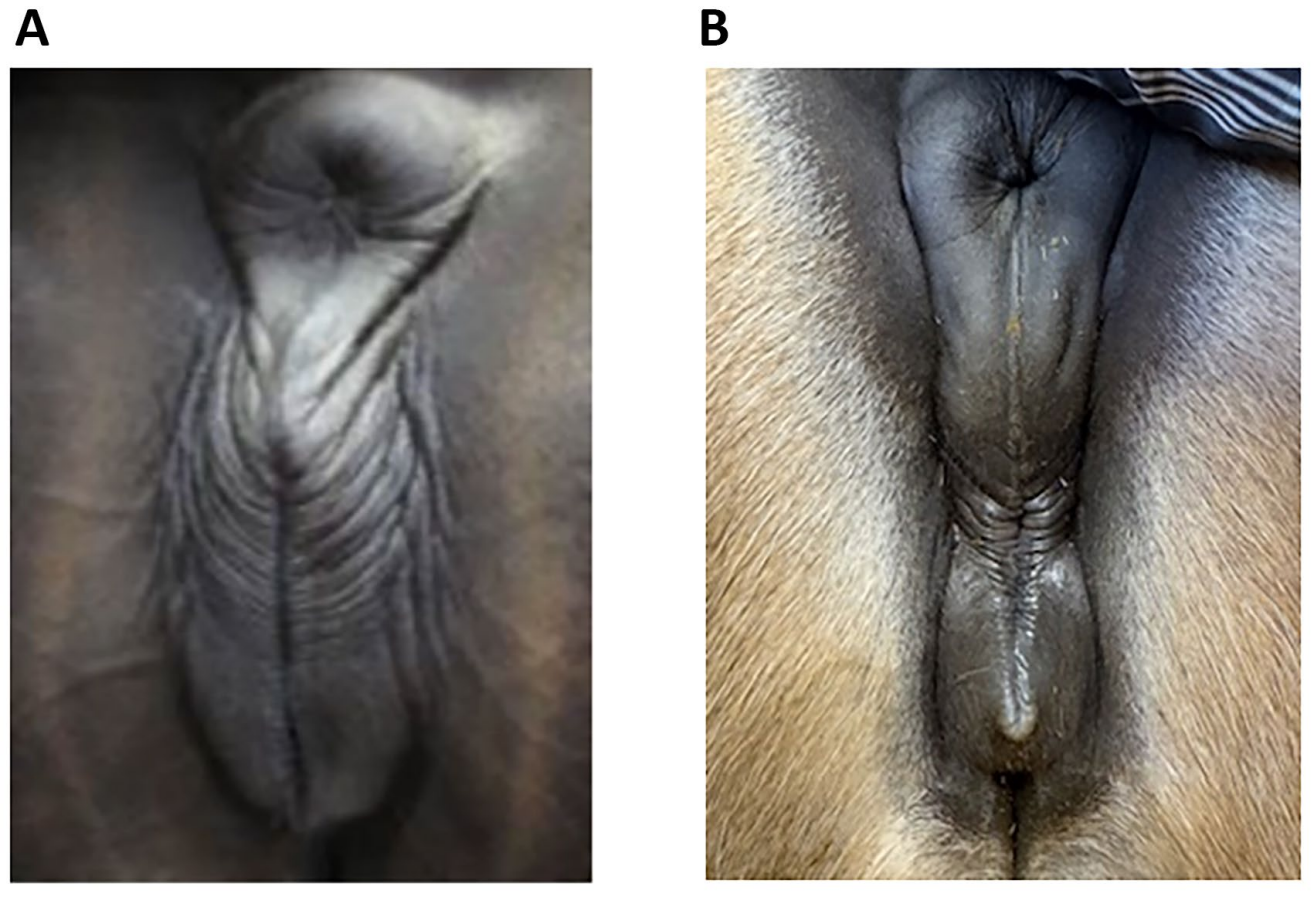
Normal vulva in late pregnant mares; note the regularly spaced, even plicae around the vulva. There was no udder activity and no discharge in these mares but **(A)** (Case 5, day 296) had an increased Combined Thickness Uterus Placenta (CTUP), which was not caused by an ascending infection. **(B)** An 8-year-old North Swedish Draught Horse at day 255 of gestation, with no udder activity, no discharge and no increased CTUP. She has not foaled yet and is therefore not included in the case report.

Insufficient sealing of the vulva can cause this problem since, in late pregnancy, the foetal weight can become heavy enough to render the vulval seal inadequate in some mares, allowing the ingress of bacteria. Figure 1 shows some examples of an inadequate vulval seal while a normal vulva is shown in Figure 2. The incidence of placentitis is estimated to be between 3 and 7% (2). Most studies on placentitis in mares are induced rather than spontaneous and have been shown to be associated with uterine production of prostaglandins (PG) PGE2 and PGF2α. These can promote myometrial contraction and are a risk factor for premature parturition. However, as there is a difference in the reaction of inflammatory blood parameters in induced placentitis compared to naturally occurring placentitis (6), there is a need for reliable diagnostic blood parameters for this condition.

The clinical signs of placentitis were described previously (5). Premature udder development and/or vulval discharge are regarded as indications for further investigation. In previous publications, udder development is not mentioned as a consistent sign of placentitis (2). If the influx of pathogens overcomes the natural barriers, an inflammatory reaction will ensue, (ascending placentitis), with or without a vulval discharge due to separation of placental tissue from the endometrium. Thus, depending on the amount of placental separation, vulval discharge may be an inconsistent finding (3).

Common diagnostic tools include an ultrasound examination of the reproductive tract and examination of the cervix with the aid of a speculum; these provide the clinician with further information on the extent of the inflammation. However, some authors advise against using the speculum in placentitis cases (2) because of the risk of introducing pathogens, while other authors suggest that it may be acceptable if specifically indicated, such as in the presence of a discharge (5). Nevertheless, examination with a speculum provides important information about the colour and the softening of the cervical portio. The healthy pregnant cervix should be closed to inhibit the entry of foreign material and pathogens (7). The impact of the pathogens may increase if no remedial action is taken, likely resulting in the loss of the pregnancy. The outcome of the pregnancy will be favourable if treatment is provided as soon as clinical signs are observed. However, many mare owners are unaware of the implications of the clinical signs, resulting in a delay before the mare is presented at the clinic, and clinicians may not be certain about which treatment to use. In general, treatment with antibiotics and anti-inflammatory agents known to cross the placenta is required; vulvoplasty (a “Caslick’s” operation) will reduce further ingress of bacteria.

Poor vulval conformation can arise from a variety of causes such as cervical fibrosis, tears or adhesions, or from anatomical defects (8). A non-sealed vulva can result in abortion of the foetus, most commonly at 9–10 months of gestation, due to the increased weight of the foetus exacerbating the poor vulval seal (9). Therefore, it is surprising that so little attention is paid to vulval conformation for those mares that develop placentitis, since correction of an inadequate vulval seal could prevent recurrence of the infection later in pregnancy. Although some articles on this subject are available, most describe induced placentitis with different diagnostic methods, e.g., (10), and medical treatments (2, 3), focusing on which treatment is the most beneficial. There are different approaches regarding the duration of treatment and which antimicrobials and anti-inflammatory medications to use, e.g., trimethoprim sulfamethoxazole (TMS), alone or in combination with potassium penicillin G and gentamicin, or ceftiofur. Apart from TMS, these antibiotics have poor or no penetration into the foetal compartments; TMS has an allantoic fluid concentration equal to the concentration in serum (3). Of the anti-inflammatory medications, non-steroidal anti-inflammatory drugs such as flunixin meglumine or phenylbutazone are not known to penetrate into placental fluid. Other NSAIDs that cross the placenta are acetylsalicylic acid and firocoxib (3). Synthetic progesterones, such as altrenogest (Regumate; Merck, Sharpe and Dohme Animal Health), alter the immune system systemically and locally within the endometrium (3). These articles do not mention the importance of the vulval seal; a defective vulval seal could be the main trigger for inducing placentitis.

The aim of the present report is to provide practitioners with detailed clinical examples of the diagnosis and management of late-term equine placentitis in the field, presenting with early development of the udder and/or discharge from the vulva. The treatments chosen in each case represent the best practice at the time, and changed somewhat over time, reflecting increasing knowledge of drug pharmacodynamics in the uterus.

## 2 Diagnosis and treatment

The cases described here were mares with ascending placentitis, referred to Haddebo Seminstation, Sweden, over a 12-year period (2012–2024). The 17 mares comprised 15 Swedish Halfbred and 2 Icelandic horses. Seven of the mares were old maiden mares (mares that were >8 years old and had never had a foal). Four had previously aborted their first foetus and were now pregnant for the second time. The remaining six animals were mares that had previously foaled. Their ages varied from 8 to 20 years. A summary of each case is provided in Table 1.

**Table 1:** Summary of mares presenting with ascending placentitis in chronological order from 2012 to 2024: clinical signs, treatment and outcome.

Case	Age of mare (years)	Gestation length	Anamnesis	Udder activity	CTUP mm	Discharge	Treatment	Day foaling	Foal status	Placenta
1	8	311	F	++	CTUP not known, detached a-c	++	TMS Caslick	316	Meconium retention	Ret sec, patches of necrotic villi throughout the entire body of the placenta, cervical star demarcation zone of the location of detachment
2	12	270	F	+++	17	+	TMS iv NSAID Ventipulmin Caslick extended	333	Sepsis+ Plasma Low IgG	Several detached areas with blood clots located at placenta body
3	15	1. 265 2. 295	F, aborted first foal previous year	1. +++ 2. 0	1. 7 2. 22 3. Large detached a–c	1. +++ 2. +	TMS, NSAID Ventipulmin, Caslick extended	305	Sepsis+++	30% body necrotic villi mucous pus smear, demarcation zone, funisitis
4	13	1. 288 2. 296	OM	++	1. 7 2. 10 detached a–c	NAD	TMS, NSAID Caslick extended	323	Sepsis++	Cervical star weak demarcation zone, surface mucous pus
5	13	296	F	+++ unweaned foal	11	NAD	No treatment	333	Dysmature, Plasma low IgG	Lymphatic oedema placenta, umbilical cord 145 cm
6	9/10	1. 120, 2. 274	OM	2. +	1. 9 2. 14	NAD	1. TMS Caslick 2. TMSX1	329	Normal	NAD
7	13	256	OM	++	14 detached a–c	++	TMS ASA Caslick	342	Sepsis +	Ret sec, cervical star oedema necrotic villi
8	16/17	1. 65, 2. 175, 3. 301	F, d 28 poor blood circulation and a thin umbilicus, d135 amnion fluid patches	++ d 301	1. 8, 2. 13, 3. 9.5	NAD	TMS ASA d 65–142, Caslick extended d175–325	325	Sepsis, plasma	Uterene wall very oedematous
9	12	294	OM	+++	14.5	NAD	TMS ASA Caslick	321	Normal plasma low IgG	NAD
10	11	315	F	+	11.05	++	TMS ASA Caslick	332	Normal plasma low IgG	NAD
11	20	180	F, first foal. Septic foal that died two years previously	NAD	4.7	++	TMS Caslick	328	Normal	NAD
12	13	282	F, First foal “red bag” with dead foal 2 yrs. before	++	10	NAD	TMS ASA Caslick	326	Normal, plasma	NAD
13	17	262	OM	++	15	NAD	TMS ASA Caslick	320	Meconium retention	NAD
14	15	0.300	F	++	10–14 detached a–c	NAD	TMS ASA Caslick	322	Normal	NAD
15	16	251	OM	+	11	++	TMS ASA Caslick extended	324	Normal	NAD
16	14	310	F Aborted first foal 2 years previously	+++	17	+	TMS ASA Caslick	333	Normal plasma	NAD
17	17	302	F	++	11.5	NAD	TMS ASA Caslick	345	Normal plasma	NAD

Gestation length: days of pregnancy at examination.

OM = Old maiden mare, F = mare at foot.

Udder activity; + = teats swollen, ++ = teats and udder swollen, +++ = full udder, milk; NAD = no abnormality detected.

CTUP = combined thickness uterus and placenta (mm); detachment of allanto-chorion (a–c) is noted.

Discharge: + sparse reddish discharge, ++ moderate reddish discharge, +++ profuse discharge. 0 = no discharge.

Treatment: TMS = trimethoprim sulphate, NSAID = non-steroidal anti-inflammatory drug; Flunixin meglumin, ASA = acetylsalicylic acid, Caslick = vulvoplasty.

Foaling days: gestational day when the mare foaled.

Foal status; ++ = moderate-severely affected, foal cannot keep a normal feeding schedule, moderate-high inflammatory blood results.

+++ very sick foal, needs help to stand up, cannot keep normal feeding times, high inflammatory results.

Placenta: Ret sec = retained placenta, the mare did not expel the placenta within 3 h postpartum, 0 = normal placenta.

All the mares presented with either premature mammary activity and/or vulval discharge. The vulval discharge was observed with placenta detachment and appeared as dark blood-stained discharge, either as crystals on the vulval lips or dripping out through the vulva, depending on the amount.

On arrival, a thorough examination of the vulva was carried out to assess its capacity to seal properly. The apposition of the vulval lips was regarded as if it were a zip. If there was any discontinuity in the lips of the vulva, i.e., in the edges of the vulval lips at the pelvic brim or folds at the edges, a deficient vulval closure was suspected, which was likely to allow the ingress of bacteria (Figure 1).

A gynaecological examination was performed to investigate the closure of the cervix and to provide a measure of the combined thickness uterus placenta (CTUP) using ultrasound (11, 12). In mares with a soft cervix and increased CTUP for their gestational time, the cervix was examined using a speculum (single-use speculum; Minitüb; Tiefenbach, Germany) after first washing the vulva thoroughly with betadine soap and using a sterile vaginal cream. The appearance of the portio was noted; in some cases, it was reddish and slightly open instead of pale and sealed. The ultrasound examination also revealed the structure of the chorio-allantois and any detachment, the presence of a live foetus, and the appearance of the amniotic fluid. After 7 months of gestation, the CTUP is expected to be no more than 1 mm less than the month of pregnancy; i.e. if the mare is 9 months pregnant, the CTUP should be 8 mm (5).

## 3 Individual case histories

The cases are presented in chronological order. For the earliest cases, no recommended evidence-based treatment protocols were available. Cases 1–4 were treated with trimethoprim/sulfamethoxazole 400/80 (TMS) intravenously at 15–30 mg/kg twice daily (Intervet/Schering-Plough Animal Health). Cases 2–4 were also given NSAID (Cronyxin; Bimeda; UK) 100 mg/mL, Flunixin (Rakshit, India), 5–10 mL intramuscularly once daily.

### 3.1 Case 1

An 8-year-old Icelandic mare had foaled the previous year but had been difficult to get in-foal again because of endometritis. The mare was presented at 311 days of gestation. There was a small amount of bloody discharge from the vulva, and a full udder except for the teats. Placental detachment was evident on ultrasound examination; upon viewing with a speculum, the cervix appeared red and was not properly sealed. The mare was given TMS intravenously for 1 day, and thereafter the owners continued oral treatment twice daily until foaling. Pneumovagina was present; therefore, a vulvoplasty was performed, despite being so close to parturition. The mare started to produce milk on day 315 of gestation and foaled the next day. The placenta was retained. The body of the placenta had patches of atrophic villi, and the detached area was clearly visible close to the cervical star. The foal was bright and alert, with a Foalcheck score of >>8 g/L (SNAP Foal IgG, Idexx Laboratories, Holland). However, it needed an enema for meconium constipation.

### 3.2 Case 2

This 12-year-old mare was presented at 270 days of gestation. She had respiratory problems due to sensitivity to being indoors. She was given additional Clenbuterol (Ventipulmin; Boehringer Ingelheim Animal Health) to facilitate her breathing and to avoid contractile activity in the uterus. The blood work showed the following results: SAA (<5 mg/L), WBC (8.2), and fibrinogen (3.2 g/L), all of which were within normal values. The CTUP was 17 mm, and her udder was active. She was given TMS twice daily intravenously and NSAID intramuscularly (Flunixin). Administration of Flunixin was stopped at 315 days as the mare was becoming more sensitive to injections. There was no total regression of udder activity, and there was a small amount of dark bloody discharge from her vulva. Two days before foaling, the udder was leaking milk. The placenta had two detached areas, one close to the cervical star and the other at the base of the pregnant horn. The umbilicus measured 95 cm. At birth, the colostrum had a low IgG level (40 g/L); the foal was lethargic, the mucous membranes were slightly injected, but the blood work was within normal values (WBC 8,000, fibrinogen 2.7 g/L, SAA < 5 mg/L, Foalcheck 6 g/L). An indwelling catheter was sutured into the jugular vein. The foal was given 1 L hyperimmune plasma and antibiotics (benzyl penicillin four times daily and gentamicin (Ceva Animal Health) once daily for 4 days). At this time, the umbilicus was dripping urine and was swollen, and the foal’s temperature was increasing. Therefore, the antibiotic was changed to ceftiofur for 10 days, with daily washing of the umbilicus with chlorhexidine. Bacteriological culture from the umbilicus indicated the presence of *Staphylococcus hemolyticum*, which was sensitive only to enrofloxacin. At the time of the bacterial result, the umbilicus was almost dry. All antibiotics were stopped as the blood results were within normal values, although local treatment with chlorhexidine was continued for a further 2 days.

### 3.3 Case 3

**Figure 3:**
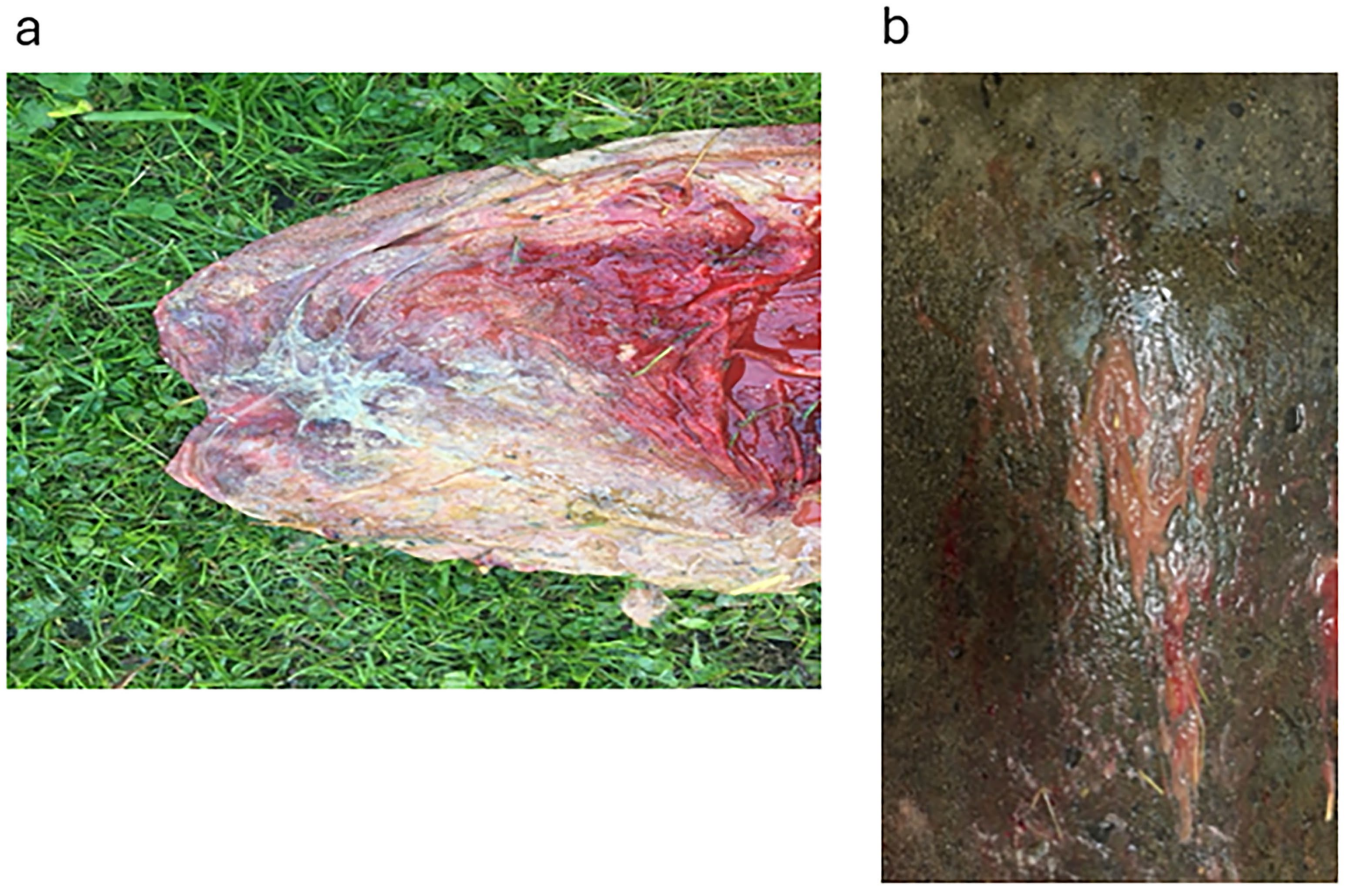
Placenta from Case 3. **(a)** Shows the cervical star and its surrounding area, the effect of the necrotising inflammation of the chorioallantois and the demarcation zone to somewhat more healthy-looking tissue. **(b)** Shows the thick mucus exudate that covered large areas of the chorion.

During pregnancy in the previous year, this 15-year-old mare produced a bloody discharge from the vulva, culminating in the loss of the foal. She was presented at 265 days of the current gestation with bloody fluid issuing from the vagina. Examination with a speculum revealed an inflamed cervix exuding reddish fluid. The udder was active, but it was cold and floppy. Treatment with TMS x2 intravenously was continued for 31 days, with an additional treatment of benzyl penicillin 3 g and gentamicin 100 mg/mL (10 mL mixed in 100 mL sterile water), infused into the vagina. Flunixin (Cronyxin 10 mL) was administered intramuscularly for 10 days, 5 mL for 15 days, and 3.5 mL for 6 days; in total, the treatment was given for 31 days. After 2 days, the udder and vulval discharge regressed; on the 4th day of treatment, there was no vulval discharge and the udder was less tense. However, the clinical signs resumed after 2 days. The vaginal infusion was then stopped. In addition, clenbuterol was also administered orally twice a day for 31 days. The blood work showed a white blood cell count (WBC) of 7.2, differential neutrophils/lymphocytes 5.0/1.6, SAA < 5 mg/L, and fibrinogen 2.2 g/L. On ultrasound examination, there was a large area (20 cm) of placental detachment, but the foetus was lively. On examination with a speculum on the 8th day after treatment started, the cervix was pink and closed, with no bloody discharge. On Day 14 after treatment started, the udder had regressed completely and there was no discharge from the vulva. However, on ultrasound examination on Day 26, the CTUP was 22 mm, and the foetus was not as active. Pus was noticed from the vulva on Day 25, with the bacterial growth identified as *Enterococcus faecalis* and yeast. By Day 28, the udder was developing normally, and no pus was seen from the vagina. On Day 30, procaine penicillin (Ethacillin vet) was administered intramuscularly for 12 days. On Day 34, there was pus coming from the vagina again; by Day 40, pentoxifylline (Trental, no longer available) was added to the treatment, given orally at 10 tablets twice a day. The mare foaled the same day at 305 days of gestation. The entire placenta was hyperaemic and 30% of the placental body was covered in mucus; there was an abundance of thick, necrotic villi and a sharp demarcation zone to the more normal placenta (Figure 3a); the umbilical cord was thick and hyperaemic. The villi of the placenta were covered with a thick mucus smear (Figure 3b).

The foal was very weak, with violet–blue mucous membranes, opisthotonic seizures and omphalitis. An intravenous catheter was sutured into the jugular vein; the foal was given 5 mg/kg Ceftiofur (Excenel; Zoetis Sweden) twice within 10 min after birth and 1 L of hyperimmune plasma within 40 min after birth, as the colostrum IgG was only 30 g/L. The foal was fed milk from a bottle every 20 min to encourage the suckling reflex. The foal drank readily and gained strength quickly, being able to stand by itself after 12 h, although it could not maintain its feeding schedule. Therefore, the foal was encouraged to get up every 20 min. The Foalcheck was >8 g/L after plasma infusion, WBC 9.7, differential 7.9/1.0, fibrinogen 4.9 g/L, SAA < 5 mg/L. An ultrasound examination of the umbilicus showed thickening from the urethra to the bladder, resulting in the foal urinating small amounts frequently. On the second day, omeprazole (Gastrogard) was added to the medication list; the colour of the mucous membranes was improving to a more pinkish hue, but the foal still needed help to maintain its feeding schedule. On the third day, the foal was taken out for a short walk to improve the strength of its joints. Blood work on the third day showed that the WBC was 10.4, differential 8.5/1.3, fibrinogen 4.3 g/L, SAA < 5 mg/L. A foul-smelling diarrhoea started on the fourth day, with the foal being alternately lethargic and very bright. The diarrhoea was treated with Diarsanyl (oral paste; CEVA, France), along with intravenous Ringer’s acetate and glucose according to the packed cell volume (PCV) and blood glucose level.

On the 7th day, gentamicin (100 mg/mL) at 6.6 mg/kg was added to the treatment. The blood work showed WBC 10.5, differential 7.6/1.8, fibrinogen 5.4 g/L, SAA < 5 mg/L, and creatinine 85 μmol/L. The culture from the umbilicus showed growth of *Staph vitulinus*, *Staph hemolyticum*, and *Enterococcus faecalis*. On the 8th day, the umbilicus started to drip urine again, but the diarrhoea had stopped. On day 10, the umbilicus was still swollen and dripping urine. The intravenous treatment was changed on day 11 to Clarithromycin (Klaritromycin tablet; Orion Pharma, Sweden) 500 mg twice daily and Rifampicin (Rimactan; Sandoz, Denmark) 600 mg once daily per orally for 8 days, according to the bacterial sensitivity. The swelling of the umbilicus decreased significantly within 2 days. Blood work showed WBC 11.6, differential 8.0/2.7, SAA < 5 mg/L, and fibrinogen 3.2 g/L. The amount of omeprazole was kept the same in order to decrease the dose per kilogram as the foal’s weight increased. By day 19, the foal was judged to be healthy; at this time, the WBC was 9.1, differential 6.0/2.4, fibrinogen 3.1 g/L, and SAA < 5 mg/L.

### 3.4 Case 4

A 13-year-old mare had an active udder at 288 days of gestation and was treated with NSAID Flunixin (Cronyxin) once a day and TMS twice a day. The udder regressed 5 days after treatment started. The Flunixin was given for a total of 16 days, at a high dose (10 mL) only on the first day and thereafter at a lower dose (5 mL) for 13 days, followed by 3.5 mL for the last 2 days. The TMS 400/80 intravenously was continued for 23 days. At foaling, the mare showed poor abdominal contractions. The foal was born in a twisted position, was cold and almost lifeless, with injected mucous membranes. It was treated with Ceftiofur (Excenel injectable; Zoetis Sweden) at a dose of 10 mg/kg twice a day, with the first injection being given <10 min after birth, and with plasma administered within 40 min after birth. The Foalcheck showed >>8 g/L, WBC 17.4 with a differential of neutrophils at 16.1, SAA 282 mg/L, and fibrinogen 2.9 g/L. The suckling reflex was poor initially but became stronger after 2 h. The foal’s posture was initially hypotonic, and it needed assistance to rise. After 12 h, it could get up with help to maintain 20-min feeding intervals, and it was observed nursing well. It was given 400 mg DMSO in 1 L of 5% glucose on the first and second days of life, omeprazole orally (Gastrogard oral paste; Boehringer Ingelheim, Germany) for 10 days, and diarsanyl 2–3 times a day for 7 days. On the third day, it was taken out to pasture; it could rise unaided and maintained its feeding times every 20 min with the help of the mare. The foal was much brighter and more alert. On the fifth day, it was coughing and had a fast respiratory rate. Ultrasound examination of the chest revealed three broken ribs, although no pneumonia was detected, with respiratory crackles only from the trachea. The SAA was 399 mg/L. A tracheal wash was performed. The antibiotic was changed to benzyl penicillin (Sandoz GmbH) four times a day and gentamycin once a day intravenously for 2 days until the results of the bacteriological culture were ready, showing a pure culture of β-haemolytic *Streptococcus*. Treatment continued with procaine penicillin (Merck, Sharpe and Dohme Animal Health) at a dose of 300 mg/mL, 3.5 mL once a day for 6 days intramuscularly. The increased respiratory rate was judged to be caused by the broken ribs. The foal was given box rest with the mare for 3 weeks. All treatments were finished on day 11, as the blood work was normal at that time.

### 3.5 Case 5

**Figure 4:**
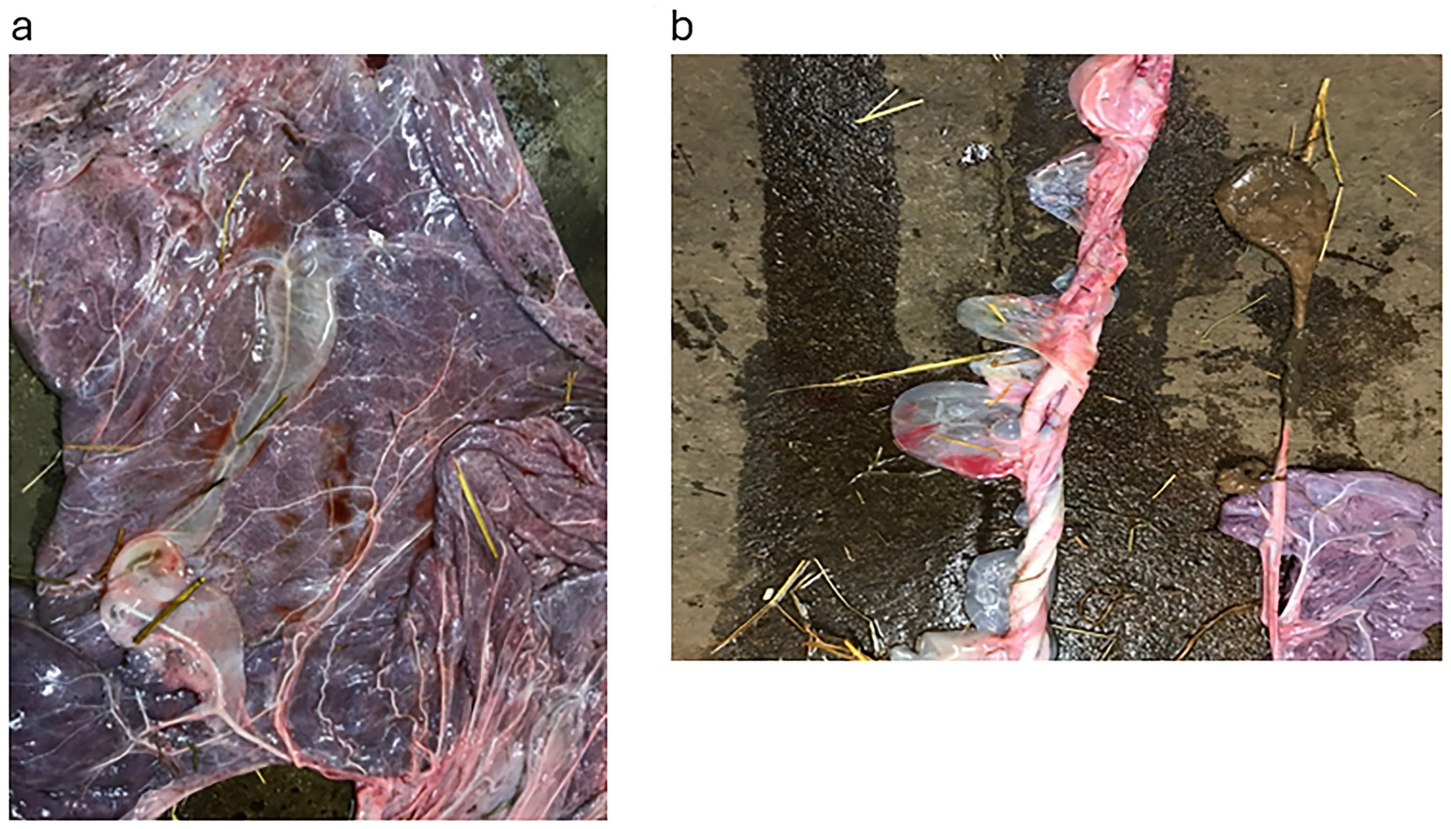
The placenta from Case 5. **(a)** Shows the extra vascular fluid around the placenta vessel on the allantoic surface. **(b)** Shows the umbilical cord, which was 145 cm and also had large pockets of extra vascular fluid.

This 13-year-old mare arrived at 296 days of gestation for foaling. She had milk in the udder, and the owner was unsure when the previous year’s foal had been weaned. On ultrasound, there was oedema in the placenta, CTUP 11 mm; the cervix was hard and closed, and the vulva was sealing very well. Since the timing of weaning was unknown, it was decided to wait to see if the udder would regress naturally, which occurred within 3 days. The mare foaled at 333 days. The placenta was excessively oedematous, with large patches of fluid outside the vessels, both on the placenta itself (Figure 4a) and on the umbilical cord (Figure 4b). The umbilical cord was 145 cm, which could have caused the oedema of the placenta (Sandra Wilsher, personal communication to EH). The foal was dysmature, with yellowish mucous membranes, and the joints on both front and hind legs were weak. The foal needed help to get up. It nursed very hungrily. The mare did not have any colostrum, producing only thin milky fluid (40 mg/mL); therefore, the foal received an infusion of hyperimmune plasma, S-LD 3.5 mmol/L. The foal was given TMS intramuscularly for 3 days. By Day 3, the S-LD was 1.2 mmol/L, and the foal was alert and judged not to require further treatment.

### 3.6 Case 6

A 9/10-year-old maiden mare presented at day 120 of gestation. Upon arrival at the clinic, the mare had a swollen vulva with faecal traces inside the vagina. There was no udder development, but some ventral oedema close to the udder; CTUP was 9 mm, with separation of the placenta ventral to the cervical star. A vulvoplasty was performed, and oral antibiotic treatment with TMS twice daily (Elanco, Denmark) was initiated. The mare returned for a check-up after 12 days. The vulva and the ventral oedema had disappeared; the CTUP was 5.4 mm, and the vulvoplasty was elongated at this time. Three months later, at 274 days of gestation, the mare kicked the owner badly and was subsequently given TMS only once daily until foaling. Udder development prior to foaling proceeded normally. The mare foaled at day 329, with a healthy foal.

### 3.7 Case 7

**Figure 5:**
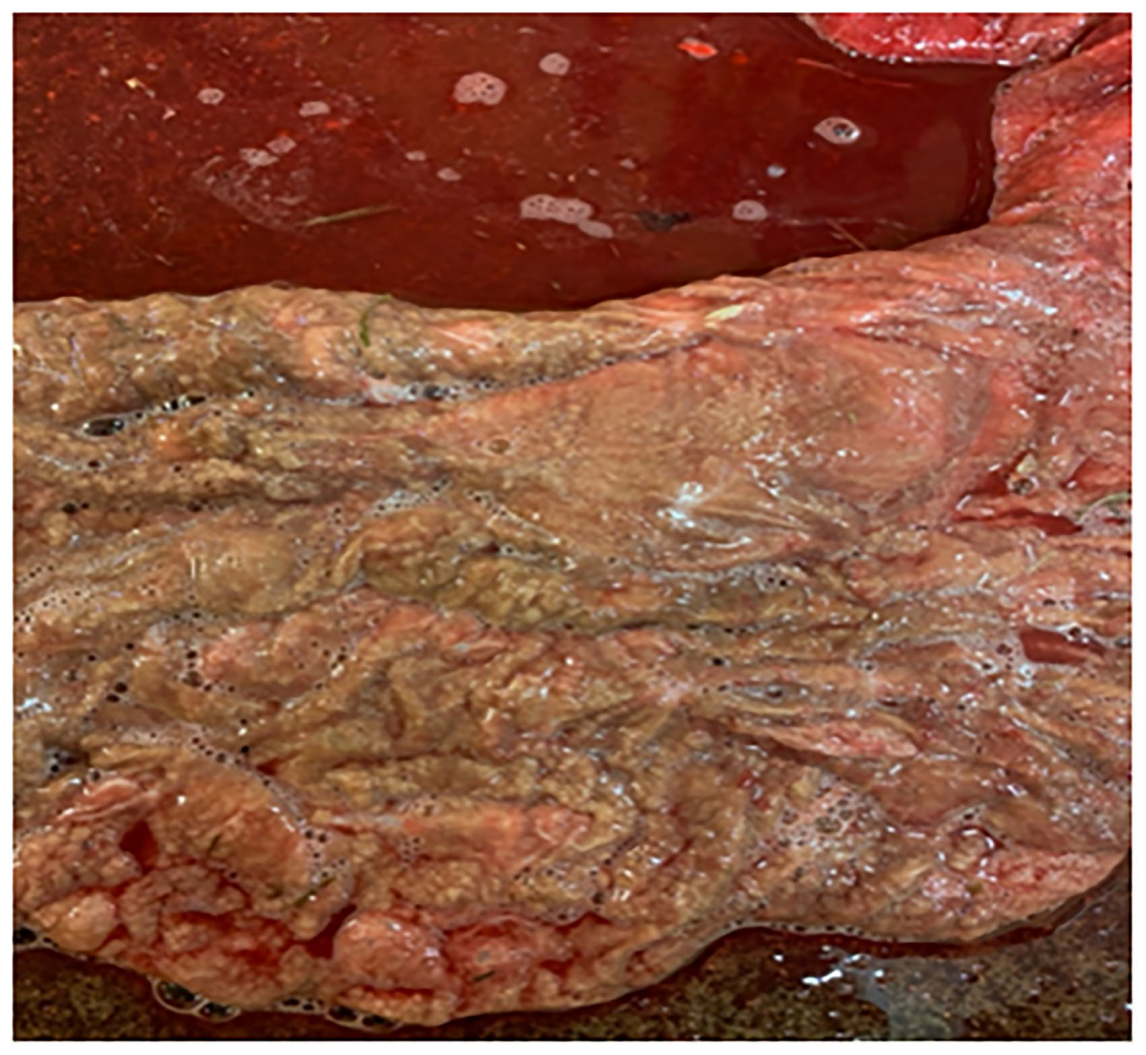
Placenta from Case 7, showing the chorionic side with an abundance of necrotic villi which covered a large portion of the placenta.

A 13-year-old maiden mare was presented at day 256 with a full udder (3+), including the teats, which had developed over the last month, and a red discharge from the vulva. On ultrasound, the CTUP was 14.3 mm, there was ventral detachment of the placenta and allantoic folds, and the cervix was not properly closed and was soft. A vulvoplasty was performed, and treatment was initiated with oral TMS twice daily. Two weeks later, there was no discharge from the vulva, and the CTUP measured 18 mm close to the cervix, although it was only 8 mm ventrally; the cervix was closed, and the allantoic fluid contained flares. Acetylsalicylic Acid (ASA; Bamyl, tablets, DrPfleger Arzneimittel GmbH, Germany) was added to the treatment 10 g twice daily orally, together with TMS until foaling. The udder was not tense but had increased in size. At 305 days, the mare returned to the clinic to stay until foaling; the CTUP was 7 mm, the cervix was softer, and there was a sparse bloody discharge. One week later, at 312 days of gestation, the CTUP was 9.8 mm, the allantoic folds could not be seen, the cervix was closed, and the udder had almost completely regressed. Two weeks later, at day 326 of gestation, normal pre-parturition udder development began. She foaled at 342 days. The placenta was retained, the cervical star was thick, and the body and pregnant horn showed abundant necrotic villi (Figure 5). The foal was able to stand within 1 h and nursed in <2 h, but it was unable to maintain a normal feeding schedule after 6 h and was lethargic. The Foalcheck result was >>8 g/L, S-LD 7.2 g/L, SAA < 5 mg/L, WBC 15,300, and fibrinogen 1.8 g/L, indicating septicaemia. The mucous membranes were pale greyish. An indwelling catheter was sutured in place, and antibiotics (benzyl penicillin four times and gentamicin once) and hyperimmune plasma were administered. The foal appeared brighter after 2 h. By the second day, the foal was extremely hungry, and on the third day, it could maintain its feeding times (three times per hour). The blood work showed SAA < 5 g/L, WBC 9.4, differential 6.3/2.6, and fibrinogen 2.8 g/L. The antibiotic treatment was concluded after day 5.

### 3.8 Case 8

A 16/17-year-old mare with a history of being barren for 5 years was diagnosed with pituitary pars intermedia dysfunction (PPID; previously known as Cushing’s syndrome) at another equine clinic and was treated with the dopamine agonist Pergolide (Boehringer Ingelheim Animal Health, tablet 1 mg). The mare had endometritis at that time and required treatment to become pregnant. She was then presented at the author’s clinic at day 28 of gestation. The umbilical cord was considered to be thin, and treatment with TMS once daily was initiated until day 40; at day 65, the placenta and the amnion wall were oedematous with folds. The TMS treatment was increased to twice daily and prolonged. By day 91, the cervix was tight, and the foetus size was within the normal parameters of a 90-day pregnancy. Treatment was supplemented with TMS and ASA and continued for another 3 weeks; it was stopped at day 111. The mare returned for a check-up at day 142, at which stage the CTUP was 4 mm, and the cervix was closed. When the mare returned 6 weeks later (day 182), the CTUP was 13 mm, there was oedema around the cervix, the cervix was soft, and there were moderate flares in the amniotic fluid. Treatment with TMS twice daily and ASA twice daily was initiated again. Three months later (day 272), she was presented for a check-up; the CTUP was 8.9 mm, there were no flares in the amniotic fluid, and the cervix was closed. Treatment was continued until she foaled. Udder development started at 303 days, at which time the CTUP was 9.5 mm. The mare foaled at 329 days. The endometrium was heavily oedematous after foaling. The foal’s mucous membranes were brick-coloured, and it was lethargic; sepsis was suspected. An indwelling catheter was sutured into the jugular vein; antibiotic treatment with crystalline penicillin four times daily and gentamicin once daily was started, and 1 L hyperimmune plasma was given. The Foalcheck result was 8 g/L, S_LD 3.3 mmol/L, which is above the normal value (1.2–2.6 mmol/L). Since the foal was born during a weekend, other bloodwork was postponed until the beginning of the following week. Two days later, the S-LD was 1.2 mmol/L, WBC was 6.3, diff 4.9/1.2, fibrinogen 3.1 g/L, and SAA < 5 mg/L. The foal was able to keep his feeding times and was alert. The antibiotic treatment was stopped after 5 days. The foal had a loose stool and was therefore given omeprazole paste for 5 days.

### 3.9 Case 9

A 12-year-old mare was presented at 294 days of gestation with a full udder and teats, with crystallised fluid at the end of the teats. On ultrasound, the CTUP was 14 mm, and the cervix was not closed. A vulvoplasty was performed, and TMS x2/day and AS Ax2/day were administered until foaling. Three days later, the udder was dripping milk. The mare foaled at 321 days, producing a healthy foal. The Foalcheck was <<8 g/L according to the attending veterinarian, and the foal was therefore given 1 L of plasma in the jugular vein.

### 3.10 Case 10

This 11-year-old mare was presented at 315 days with swollen teats and a red mucous discharge from the vulva. On ultrasound, the CTUP was 11.4 mm, the cervix was not closed, and the right vulval lip above the pelvic rim had a tear that had not been repaired from a previous foaling. A vulvoplasty was performed, and TMS twice daily and ASA twice daily were administered until foaling. She foaled 17 days later, at 332 days. The foal was healthy, and the Foalcheck result was >8 g/L.

### 3.11 Case 11

An Icelandic mare, 20 years old at presentation, had a history of producing a septic foal 2 weeks early at an equine hospital 2 years previously, which subsequently died. The mare was presented at 180 days of gestation, with a yellow discharge from the vulva of 1 week’s duration; the CTUP was 4.7 mm, and the cervix was open towards the portio. On examination with a speculum, the vagina was inflamed, and the cervix was open 1(+); at the cervix, there was marmorated sticky secretion. There were old tears on both edges of the vulva. The secretion was sampled and sent to a certified laboratory, where a small amount of growth of *Enterococcus faecalis* was found. A vulvoplasty was performed, and treatment with TMS x2/day was initiated. The mare returned for a check-up 3 weeks later, at day 188 of gestation; the vulvoplasty needed elongation. The CTUP was 5.7 mm; on examination with a speculum, the cervix was closed, and there was no secretion. The owner preferred to administer TMS x1, but 4 days later, the mare had a yellow vulval discharge again, and TMS was increased to x2/day. The mare foaled at day 328 of gestation. The Foalcheck result was >>8 g/L, WBC 8.2, differential 5.5/2.4, SAA < 5 g/L, fibrinogen 2 g/L. The foal was healthy.

### 3.12 Case 12

A 13-year-old mare presented at 282 days of gestation with a swollen udder (except for the teats) of 1 day’s duration. Her first foal, 2 years previously, had been a “red bag” delivery, i.e., premature detachment of the placenta. The foal died shortly after birth; the mare was treated for endometritis due to *Escherichia coli* infection, and vulvoplasty was performed. She was barren that year. Now, she was showing similar clinical signs as in the previous pregnancy. On ultrasound examination, the CTUP was 10 mm and the cervix was soft. Treatment with TMS twice daily and ASA twice daily was started. The vulvoplasty was extended. When re-examined at 303 days of gestation, the udder was much smaller but still active, and the CTUP was 12 mm. Ten days later, the udder was secreting transparent fluid, the CTUP was 10 mm, and the cervix was now closed. The mare foaled 13 days later at 326 days of gestation. The placenta had necrotic villi. The colostrum IgG, measured using a Brix refractometer, correlated to an IgG concentration of 28 g/L, which is regarded as an adequate level (13). The foal was bright and alert. After plasma infusion, the Foalcheck was >>8 g/L, WBC 8.4 diff 5.6/2.4, fibrinogen 2 g/L, SAA 7 mg/L. The foal remained healthy.

### 3.13 Case 13

A 17-year-old maiden mare was presented with an active udder; the CTUP was 15.7 mm, there was oedema at the entrance of the allantois, and the cervix was soft. On examination with a speculum, the vagina was red, and the cervix was open (2+). A vulvoplasty was performed, and the mare was treated with TMS twice daily and ASA twice daily until foaling. Three weeks later, the udder had regressed, and there was no vulval discharge. The mare produced a dysmature foal at 320 days. The foal had poor muscle development, weak fetlocks, an umbilical hernia, and meconium constipation. The Foalcheck result was >>8 g/L, SAA 38 mg/L, WBC 8.3 diff 65.5/1.4, fibrinogen 2.8 g/L. The constipation was treated with an enema of acetylcysteine, baking soda, and water. The foal remained healthy.

### 3.14 Case 14

This mare, 15 years old, had produced a stillborn foal the previous year, which was revived after intensive resuscitation. The mare arrived at day 300 of the current gestation, with a cold and active udder. There was a CTUP of 14 mm with oedema and ventral separation of the placenta. On examination with a speculum, the cervix was closed but had a red colour. Treatment of TMS twice daily and ASA twice daily was administered until foaling. A week after starting the treatment, the udder had regressed completely. The mare foaled a healthy foal at 322 days. The Foalcheck result was 8 g/L, and all blood parameters were normal: WBC 12,200, SAA < 5 g/L, and fibrinogen 2.2 g/L.

### 3.15 Case 15

A 16-year-old maiden mare that had previously received a vulvoplasty was presented at day 251 with a bloody vulval discharge and an active udder; the CTUP was 11 mm. On ultrasound examination, the cervix appeared tight but was not closed when examined with a speculum. The vulvoplasty was extended. The mare was treated with TMS x2/day and ASA x2/day until she foaled at 324 days. The foal was healthy at birth; the Foalcheck result was >>8 g/L, WBC 9.1, differential 7.8/0.9, fibrinogen 2.4 g/L, SAA > 5 mg/L.

### 3.16 Case 16

A 14-year-old mare, which aborted a dead foal 2 years previously, was presented at day 310 of gestation, with a well-filled udder and small crystal drops around the vulval edges. The owner reported that the mare had exhibited similar signs when she aborted in the previous pregnancy. The CTUP was 17 mm, with fluid accumulation ventral to the cervix. On examination with a speculum, the cervix was reddish and open. Since pneumovagina was diagnosed (Figure 4A), a vulvoplasty was performed and treatment with TMS twice daily and ASA twice daily was initiated. The udder started to regress a week after treatment began. The mare foaled at home at 333 days of gestation. The Foalcheck result was >8 g/L, but hyperimmune plasma was administered as well as antibiotics, Ceftiofur, for 6 days by the attending veterinarian due to the mare’s previous history.

### 3.17 Case 17

A 17-year-old maiden mare was presented at 302 days of gestation with an active udder of 1 day’s duration and a small amount of reddish fluid coming from the vulva (Figure 4B). The CTUP was 11.5 mm. The vulva was sutured, and treatment with TMS twice daily and ASA twice daily was initiated. She foaled at day 345. The foals’ blood work was normal; the Foalcheck result was >>8 g/L. WBC 7.5, differential 4.8/2.5, SAA < 5 g/L, fibrinogen 2.1 g/L. Hyperimmune plasma infusion was given at the owner’s request.

## 4 Summary of case histories

In these 17 cases, clinical signs were observed at an average of day 279 of pregnancy. Sixteen of the mares received trimethoprim sulphate twice daily either intravenously or orally. One difficult mare was only given TMS orally once daily. Three mares were also given NSAIDs intramuscularly once daily, and 10 mares were given acetylsalicylic acid twice daily until they foaled. The two Icelandic Horses were not given anti-inflammatory treatment at the owners’ request. For the five mares that had already been operated on, their vulvoplasty was extended. A vulvoplasty was carried out in 11 mares that had not previously been operated on. None of these pregnant mares were negatively affected by the use of a speculum for the vaginal examination, in contrast to the suggestion of (2).

One mare was not treated with vulvoplasty as her vulva had a perfect closure; her CTUP was increased for her gestational age, and her active udder was a result of a late-weaned foal the day before her arrival. This was confirmed as the correct diagnosis when she subsequently foaled at 333 days, although her placenta showed generalised oedema with free fluid around the placental and umbilical cord vessels. Furthermore, the umbilical cord measured 145 cm (Figures 3a,b). The foal was dysmature, i.e., it did not exhibit the usual behavioural patterns for a newborn foal, such as getting up within an hour, nursing within 2 h, and showing a brisk presentation at 4 h (14), but did not have an infection.

All the referred mares, except for two, foaled at more than 320 days, which is regarded as being within the normal range of foaling (15). The placenta appeared normal upon expulsion in 10 cases. One of these (as already mentioned) had an excessive amount of oedema outside the placental vessels and the umbilical vessels. Seven mares were previously foaling mares, four mares (cases 3, 11, 12, 16) had never given birth to a live foal, and six mares were old maiden mares. Of the 17 mares, 16 were observed to have some degree of premature udder development on arrival. Case 11 was the only mare without premature udder development. Five mares had fully developed udders and were producing milk. Of these, one foal had been weaned just prior to sending the mare to the clinic. Eight mares had some fluid discharge from the vulva, two of which also showed placental detachment. The discharge was reddish in all mares.

The presenting signs observed in these mares and foals are shown in Table 1. The length of pregnancy ranged from 175 to 320 days. The CTUP measured 5–17 mm (mean 13 mm), with mares pregnant at 175 days (<6 months) having a CTUP of 5–7 mm, at 255–295 days (8.5–10 months) 11–22 mm, and at 300–320 days (10–<11 months) 7–17 mm (11). Of the 17 mares, 5 had undergone vulvoplasty (Caslick), which had been performed in the autumn of the previous year at their pregnancy check. In five mares, the placentas exhibited necrotic villi and detached areas, with a demarcation zone close to the cervical star and/or oedema in the placenta.

Five foals were septic at birth, two had meconium constipation, and four mares produced colostrum with low IgG. The most septic foals were those from mares that had exhibited clinical signs of placentitis the longest before being presented and did not receive any anti-inflammatory drug that could penetrate the placenta. All five foals received intravenous antibiotics via an indwelling catheter sutured in place 20 min after parturition. They also received plasma within 40 min of birth. All five foals had a weak suckling reflex but drank readily. They were bottle-fed every 20 min and encouraged to drink as much as they wanted until they could drink unaided and nurse regularly. Ten foals were born healthy, of which four received plasma at the owner’s request. The blood results could not be obtained for two foals directly after foaling because it was the weekend, or the mares foaled at another location (cases 6 and 16) where such investigations were not performed. One foal (Case 3) showed a high level of fibrinogen within the first day, indicating that the inflammation started *in utero*, as it takes 48 h for fibrinogen to react (16). When the mares foaled over the weekend without access to a laboratory, a field device, Lactate Pro 2 (Arkray Factory Inc., Japan), was used to measure the L-lactate (cases 5, 7, 8). Increased blood or plasma L-lactate can be useful in predicting morbidity and mortality in septic foals (17).

All 17 foals survived and went home healthy; they later became riding horses. For most mares arriving with an active udder, with or without milk, a regression of udder activity was noted a week after the start of treatment.

Cases 2, 3, and 4 were the foals most severely affected by septicaemia. These were the mares presenting with the worst clinical signs. Their placentas had large areas of detachment, necrotic villi, a mucous pus smear on the surface, and a demarcation zone between the affected and healthy tissue at or close to the cervical star.

## 5 Discussion

The following reflections arose from the management of these cases and are presented here with the aim of assisting other practitioners. The purpose of the treatment was to obtain an early resolution of clinical signs to maintain the pregnancy past 320 days and to deliver a viable foal. Moreover, the aim of the vulvoplasty was to prevent the ingress of bacteria via an incompletely sealing vulva and to prevent further inflammatory reactions. The anti-inflammatory drugs that can penetrate the placenta are COX 1 (ASA) and COX 2 (Firocoxib). All the mares responded well to TMS (twice daily), despite the previous administration of other antibiotics at the time of covering or artificial insemination. None of these mares were treated with Altrogenest, although there is a recommendation to administer this product to help maintain the pregnancy (2, 3).

At the time of the first cases (2012–17), little was known among horse owners about placentitis, and these mares may have shown signs for a prolonged period before being presented at the clinic. Bernard et al. (18) also reported that mares with placentitis are often presented late in the course of the condition. Furthermore, during the earlier cases, there was a paucity of knowledge about which drugs could penetrate the placenta. Therefore, these mares were treated with a non-steroidal anti-inflammatory drug (NSAID) to complement trimethoprim sulphate (TMS), which most likely did not provide an appropriate anti-inflammatory effect. These mares foaled at 305, 316, 324, and 333 days. Their foals showed signs of prematurity with high inflammatory blood parameters and needed intensive care to survive.

Acetylsalicylic acid (ASA) was shown to be an effective COX 1 anti-inflammatory with penetration into the placenta (19). It was also shown to increase blood flow into the placenta, which could be favourable in a pregnancy in an old maiden mare. Treatment with COX 1 has been suggested for women for its vasoactive properties (19); administration to mares could reduce the risk of intrauterine growth restriction (IUGR), commonly occurring in foals from old maiden mares (20). However, ulceration in the gastrointestinal (GI) tract could be a complication of long-term medication, and uterine contractions could be negatively affected. None of the 10 mares described here showed any signs of ulcers in the GI tract during treatment and had no problems during foaling.

Some mares, which had received a vulvoplasty during the breeding season, still presented with signs of placentitis and needed an elongation of the original vulvoplasty. The mares with a shorter duration of signs gave birth to healthy foals. One mare that had milk in her udder at 10 months was shown to have weaned her foal the day before arriving, and she exhibited an increased CTUP, but her vulval conformation was perfect. This mare’s placenta was very oedematous. Therefore, it is important to inform the owner, in the case of an older multiparous mare or a high-risk mare, to observe the udder and vulva closely to detect the first signs of placentitis. If there is any early reaction of the udder or discharge from the vulva, an ultrasound examination should be implemented immediately for CTUP measurement, and the vulval conformation should be inspected.

All the mares gave birth to live foals, although some were compromised, including needing assistance to stand and nurse regularly. These foals required intensive care. However, they survived, demonstrating the importance of early antibiotic treatment and the initiation of early (and regular) bottle feeding. In all but seven cases, the placenta appeared normal on expulsion, confirming the resolution of the placentitis following effective treatment. Although difficult to prove, and therefore a limitation of this study, it is likely that these pregnancies would have been lost without treatment.

Since ascending placentitis is the most frequent cause, vulval conformation should be included in the examination of mares showing signs of placentitis. One reason for vulval conformation not being included could be because of previous recommendations to use the Caslick index (CI) when deciding if a mare required a vulvoplasty. This index is calculated from the effective length of the vulva multiplied by its angle (21). However, one study showed that even in mares with a high CI score, there were no signs of placentitis, and the CI was found to change with the body condition score (22). Therefore, a better understanding is required of which vulval conformation does not seal well enough to prevent pathogens from entering later in pregnancy. Photographs of vulvas with poor closure and vulvas with normal closure are shown in Figures 1, 2, respectively. Photograph 1:3 was taken at 264 days of gestation. Figure 2b is a normal vulva at 255 days of pregnancy for comparison.

The vulva is regarded as the first seal between the foetus and the outside world. The seal can be likened to an elastic “zip,” similar to a rubber band. The elasticity of the vulva should expand enough to allow the birth of the foal to take place and, shortly after, contract sufficiently to provide a seal against bacteria from the outside. If there is any discontinuity in the “zip,” it will not seal properly. Continuous suction due to a weakness in the elasticity of the vulva’s edge will result in a defective seal and allow an inflow of bacteria, which is the most common cause of ascending placentitis (5). This can be observed as the plicae of the edges of the vulval lips become deeper and irregularly ordered, similar to the lines around the lips of a smoker. Therefore, the CI according to Pascoe (21) may not provide sufficient indication of the potential effectiveness of vulval closure later in pregnancy (22). Furthermore, an increased CTUP as the only presenting sign is not indicative of placentitis per se. Several clinical signs taken together, such as premature udder development, vaginal discharge, and increased CTUP, are needed to make an accurate diagnosis of placentitis. However, if there is an ascending infection via the vulva during pregnancy, it is likely that the sealing of the vulva is insufficient, and an examination of vulval conformation should always be conducted to determine if a vulvoplasty is required.

Examples are presented as Figures 1a–c. The mare in Figure 1c is an 8-year-old trotting mare, at day 265 of gestation, with a CTUP of 7–11 mm, placental detachment, a soft cervix, a slight reddish discharge from the vulva, and an active udder. She was given 52 g TMS twice daily, 5 g ASA twice daily, and a vulvoplasty was performed. Figure 2b is from an 8-year-old North Swedish Draught Horse, at day 255 of gestation, with no clinical signs indicating placentitis. The vulva of the two mares is very similar in appearance, highlighting the importance of conducting a thorough clinical examination before proceeding with treatment or performing a vulvoplasty.

## 6 Conclusion

The first signs of an ascending infection and placentitis are premature udder development and/or vulval discharge. In cases with ascending placentitis, the ingress of bacteria occurs through an inadequately sealed vulva. The cases presented here illustrate the importance of early diagnosis of placentitis to achieve resolution of the infection, as well as correction of vulval conformation to establish an effective vulval seal. The latter is crucial to inhibit the continued ingress of bacteria and to prevent further infection. Therefore, examination of the vulva must be included in the initial gynaecological evaluation of a mare presenting with premature udder development and/or vaginal discharge in late pregnancy. Effective treatment of the infection can be achieved with trimethoprim sulphate twice a day and acetylsalicylic acid, a COX-1 anti-inflammatory drug, until foaling. Sickly foals respond well to intensive care. When performing vulvoplasty, clinicians should be encouraged to ensure that the resulting tissue bridge will be sufficient to provide an effective vulval seal for the entire pregnancy. The COX2 inhibitor (Firocoxib), which has been recommended recently, was not used by the author since the results with COX1 (ASA) were satisfactory, and most of the mares were older and would benefit from treatment with COX1.

## Data Availability

The original contributions presented in the study are included in the article/supplementary material, further inquiries can be directed to the corresponding author.
